# Variability in the Center of Mass State During Initiation of Accurate Forward Step Aimed at Targets of Different Sizes

**DOI:** 10.3389/fspor.2021.691307

**Published:** 2021-08-19

**Authors:** Hiroki Yamada, Masahiro Shinya

**Affiliations:** Graduate School of Humanities and Social Sciences, Hiroshima University, Hiroshima, Japan

**Keywords:** postural control, center of pressure, anticipatory postural adjustment, speed-accuracy tradeoff, motor control

## Abstract

Motor control for forward step initiation begins with anticipatory postural adjustments (APAs). During APAs, the central nervous system controls the center of pressure (CoP) to generate an appropriate center of mass (CoM) position and velocity for various task requirements. In this study, we investigated the effect of required stepping accuracy on the CoM and CoP parameters during APA for a step initiation task. Sixteen healthy young participants stepped forward onto the targets on the ground as soon as and as fast as possible in response to visual stimuli. Two target sizes (small: 2 cm square and large: 10 cm square) and two target distances (short: 20% and long: 40% of the body height) were tested. CoP displacement during the APA and the CoM position, velocity, and extrapolated CoM at the timing of the takeoff of the lead leg were compared among the conditions. In the small condition, comparing with the large condition, the CoM position was set closer to the stance limb side during the APA, which was confirmed by the location of the extrapolated center of mass at the instance of the takeoff of the lead leg [small: 0.09 ± 0.01 m, large: 0.06 ± 0.01 m, mean and standard deviation, *F*_(1, 15)_ = 96.46, *p* < 0.001, η^2^ = 0.87]. The variability in the mediolateral extrapolated center of mass location was smaller in the small target condition than large target condition when the target distance was long [small: 0.010 ± 0.002 m, large: 0.013 ± 0.004 m, *t*(15) = 3.8, *p* = 0.002, *d* = 0.96]. These findings showed that in the step initiation task, the CoM state and its variability were task-relevantly determined during the APA in accordance with the required stepping accuracy.

## Introduction

When an individual stands at the edge of a pond and tries to step on a small stepping stone in the pond, he or she needs to accurately control his or her posture and foot placement at the same time. The question posed in this study is as follows: how does the central nervous system control the movement to achieve an accurate step? Step initiation or gait initiation is the transition between a static (or quasistatic) standing posture and a dynamic stepping/walking state (Patla et al., 1993; Yiou et al., 2017). The first biomechanical feature of forward step initiation is the transfer of the center of mass (CoM) forward and toward the supporting limb (Crenna and Frigo, 1991; Lyon and Day, 1997; Massot et al., 2019). This weight transfer is accomplished by a shift in the center of pressure (CoP) backward and toward the side of the swing limb, which is often called an anticipatory postural adjustment (APA).

Through APAs, the CoM position and velocity (henceforth, we refer to the CoM position and velocity as the CoM state) must be appropriately set when the leading limb lifts from the ground because once the leading limb is no longer on the ground, the CoM motion is dominated by passive mechanics, and the CoM falls away from the supporting limb like an inverted pendulum (Lyon and Day, 1997; Pai and Patton, 1997; Hof et al., 2007). Thus, the CoM state at the instance of takeoff of the leading limb is task-relevantly controlled depending on various task constraints, such as step length (Brenière et al., 1987; Zettel et al., 2002), temporal pressure (Schlenstedt et al., 2017), step direction (Tateuchi et al., 2011), and the presence of obstacles (Yiou et al., 2016a). For understanding the mechanisms of accurate step initiation, it is essential to investigate how the CoM state and its variability are controlled in an accurate step initiation task.

A few studies have investigated the speed-accuracy tradeoff principles in forward foot-reaching tasks (Duarte and Latash, 2007; Bertucco and Cesari, 2010; Aloraini et al., 2019). In these studies, participants were asked to point to targets at various distances and of various widths with the great toe. The authors reported that the time to complete the foot-reaching task was a function of the index of difficulty: the results were well-explained by Fitts' law. They also analyzed CoP amplitude during APAs, which was scaled by the required accuracy of foot placement. It should be noted that the tasks performed were discrete pointing tasks with one foot, during which the supporting limb stayed at the initial standing posture. In addition, these studies focused on control parameters during APAs, such as CoP displacement or EMG activity. Because the human body is a physically complex system, the CoM state is determined through the complex equations of motion with the CoP parameters and duration of the APA phase. To investigate the motor control for an accurate step initiation, the CoM, which is the important controlled variable of the posture, and its variability should be analyzed as well as the CoP parameters.

The purpose of the present study was to investigate the effect of required accuracy in a forward stepping initiation task on the CoM state and its variability during APAs. We have three hypotheses. First, we expect that the time to complete the forward stepping task would be longer when the target was small. Second, we expected that the CoM would be shifted more toward the stance limb side and the stepping speed would be slow in the small target condition to prevent falling toward the side of the swing leg. Third, we hypothesized that the variability in the CoM state would be smaller when the participants were required to step on smaller targets.

## Methods

### Participants

The inclusion criteria as a participant of this study were (1) being healthy young university students, (2) having a normal or corrected-to-normal vision, and (3) being able to understand instruction in Japanese. The exclusion criteria ensured that none of the participants (1) had no history of neurological or musculoskeletal disorders, (2) need the assistance of a wheelchair or cane in daily living, and (3) had symptomatic cardiovascular diseases. Participants were recruited by using a flier. Sixteen healthy young male adults (age: 19.8 ± 1.1 years; height: 171.1 ± 6.5 cm; weight: 60.8 ± 4.9 kg; mean values and standard deviations) volunteered in the study. Note that as only males were recruited, our results did not reflect female-specific features of the behavior. The participants were fully explained about the study and provided written informed consent. The study was conducted in accordance with the Declaration of Helsinki and approved by the local ethics committee at the Graduate School of Integrated Arts and Sciences, Hiroshima University (approval number: 30-19).

### Experimental Protocol

**Figure 1:**
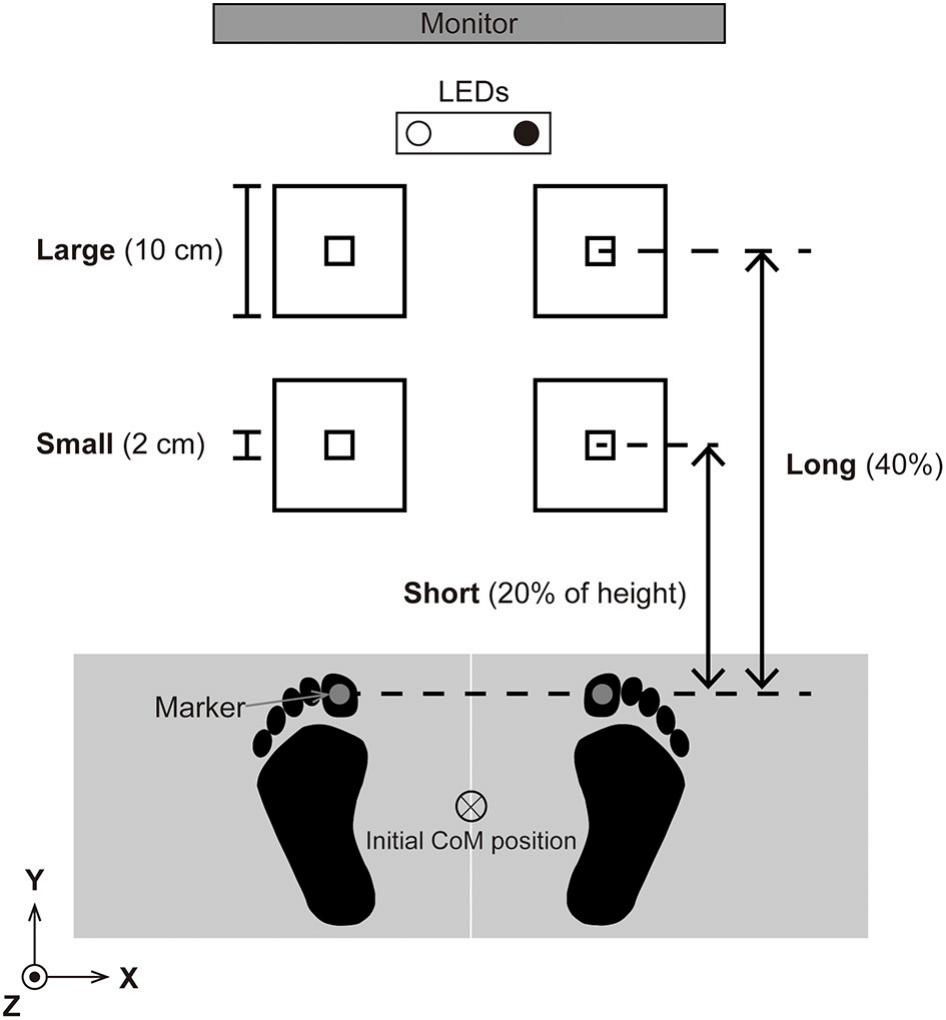
Experimental setup. The participants were asked to step forward “as soon as, as fast as, and as accurate as possible” when one of the LEDs illuminated. The left and right LEDs illuminated at random, and whether the right or left LED was illuminated informed the participants which leg should be the leading leg. We tested two target sizes, small (square 2 cm on a side) and large (10 cm), and two target distance conditions, short (20% of each participant's height) and long (40% of each participant's height). The participants repeated 40 steps for each condition (total 160 trials). The position of the center of pressure (CoP) was visually feedbacked on a monitor in real time so that the participants were able to set the CoP in the middle of the base of support.

A schematic overview of the laboratory setup is illustrated in Figure 1. Initially, the participants stood barefoot on the force platforms in an upright posture with their arms placed alongside their trunk. We instructed the participants to place their feet so that the inner edge of their feet were parallel and the inter-thumb distance was equal to the inter-acromion distance. The participants achieved the initial CoP position, which was located in the middle of the base of support, by using real-time CoP visual feedback on a monitor that was located 3.0 m in front of the participant. For the visual feedback, the CoP signal was smoothed by using 2nd order Buttherworth filter with cutoff frequency of 10 Hz. The visual feedback was programmed in LabVIEW 2017 and the latency and the refresh rate of the monitor was 5 ms, and 60 Hz. Once the initial CoP position was set, the participants were asked to look at two LEDs.

Two LEDs were placed on the floor 1.5 m in front of the participant and separated 0.3 m in the mediolateral direction. When one of the two LEDs was illuminated, the participants started the task by stepping forward. If the right LED was illuminated, the right leg was the leading leg and the left leg was the trailing leg, and vice versa if the left LED was illuminated. We instructed the participants to step as soon as, as fast as, and as accurately as possible so that both legs were on the targets marked by the tape on the floor. Note that the task included stepping with both legs, which is different from the foot-pointing tasks performed in previous studies (Duarte and Latash, 2007; Bertucco and Cesari, 2010). Although monetary reward does not necessarily have a positive effect on psychological tasks (Mobbs et al., 2009), in our preliminary experiment where we did not use monetary rewarding, some participants did not perform the task seriously. Therefore, to ensure both speed and accuracy, we told the participants that task performance was evaluated according to both speed and accuracy, and that the top six participants would receive a monetary reward of 1,000 JPY.

We tested two target size conditions and two distance conditions (i.e., four conditions in total). The target size was either small (a square with size measuring 2 cm long) or large (a 10 cm square). The location of the center of the target remained the same across the target size conditions. The target distance was either 20% (short) or 40% (long) of each participant's height. The participants repeated 40 trials for each condition. The left LED was illuminated in 20 trials of the 40, and the right LED was illuminated in the rest of the 20 trials. To prevent the participants predict which leg to step first, the order of the left and right LED illumination was randomized so that the same LED was not illuminated on more than three consecutive trials. Three familiarization trials were allowed before data were collected for each condition. The experimental design was a block design so that 40 trials (20 left and 20 right LED were pseudorandomly presented) were repeated. The order of the four conditions (ShortSmall: SS, ShortLarge: SL, LongSmall: LS, LongLarge: LL) was counterbalanced by using the Latin square. We have four sequences of conditions: SS-SL-LL-LS, SL-LS-SS-LL, LS-LL-SL-SS, and LL-SS-LS-SL. Each sequence was used for four participants. In this way, since every single condition follows every other condition once, we minimized the inter-condition carryover effects on statistical analysis.

### Data Collection

We measured three-dimensional stepping kinematics by using an optical motion caption system (Qualisys Track Manager, Qualisys, Göteborg, Sweden) with eight cameras (Qualisys-Miqus M3, Qualisys) at a sampling rate of 250 Hz. Twenty-two infrared reflective markers were placed on 22 anatomical landmarks: the left and right tragi, acromia, anterior superior iliac spines, greater trochanters, medial epicondyles, lateral epicondyles, inner malleoli, lateral malleoli, distal condyles of the second metatarsal bones, heels, and nail plates of the thumbs. The ground reaction forces were acquired using two force plates at a sampling rate of 1,000 Hz (FP4060-10-2000, Bertec, Columbus, U.S.A.). To measure the LED lighting timing, the voltage applied to the LED was also measured at a sampling rate of 1,000 Hz.

### Data Analysis

**Figure 2:**
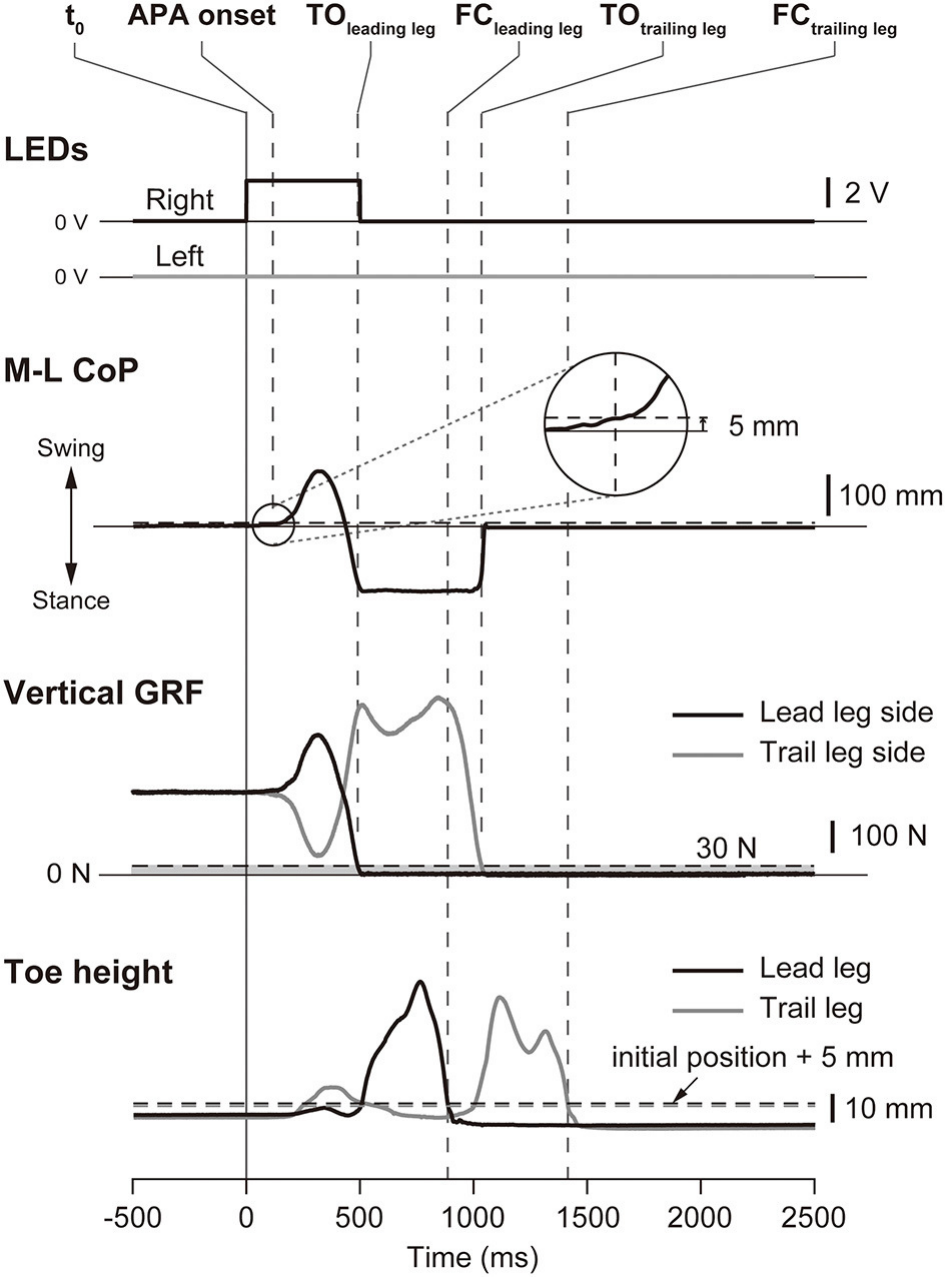
Definitions of the temporal events. The figure shows a typical example of the LED signals, the mediolateral center of pressure (M-L CoP), the vertical ground reaction force (zGRF), and the vertical position of the toes in the small target short distance condition. In this trial, the right LED was illuminated at t0 (vertical solid line), so that the right leg was the leading leg. The time of APA onset was defined as the instant when the M-L CoP deviated by 5 mm from its baseline value, the time of takeoff (TO_leading_ and TO_trailing_) was defined as the instant when the vertical ground reaction force of the leg decreased below 30 N, and the time of foot contact was defined as the first instant when the vertical position of the toe was <5 mm from the initial static posture. These temporal events are shown as vertical dashed lines. TO_leading_: takeoff of the leading leg; FC_leading_: foot contact of the leading leg; TO_trailing_: takeoff of the trailing leg; FC_trailing_: foot contact of the trailing leg.

The kinematic data and force plate data were smoothed by using a second-order low-pass Butterworth filter with cut-off frequencies of 10 and 50 Hz, respectively. The timings of the temporal events were determined from biomechanical traces (Figure 2). The time of APA onset was defined as when the CoP moved for the first time in the lateral direction by 5 mm or more from the initial position. The time of takeoff was defined for the leading and trailing legs as when the vertical ground reaction force (GRF) decreased below 30 N. The time of foot contact was the first instant when the vertical position of the toe was <5 mm above the initial position after takeoff. These thresholds for the analyses were chosen based on our preliminary experiment. The time to complete the task was defined as the time interval between the illumination of the LED and the foot contact of the trailing limb.

As Shapiro-Wilk tests revealed no violation of normality on the foot placement data for each participant, variability in the foot placement was quantified by using the standard deviation of the foot placement, which was calculated in the M-L and A-P axes and for the leading and trailing limbs. During the APA, the CoP moved backward and toward the leading leg. The CoP parameters were regarded as the control variables of the APA. The maximum CoP displacement was calculated for the anteroposterior (AP CoP) and mediolateral (ML CoP) axes. In addition to the CoP parameters, we analyzed the CoM state (i.e., the position and velocity of the CoM) at the time of the takeoff of the leading leg, which was regarded as the controlled variable of the APA. We calculated the CoM state based on a seven-segment rigid-body model including the right and left thighs, lower legs, feet, and the head, arms, and trunk were considered one segment (Dempster, 1955). The velocity of the CoM was calculated using three-point numerical differentiation. In addition to the position and velocity of the CoM, we analyzed the extrapolated center of mass (xCoM) based on a study by Hof et al. (2005) using the following equation: *xCoM* = *CoM* + *velCoM*/ω_0_, where *CoM* and *velCoM* are, the position and velocity of the CoM, respectively, and ω_0_ is the eigenfrequency of the body modeled as an inverted pendulum. This eigenfrequency was calculated as follows: ω_0_ = √(*g*/*l*), where *g* = 9.81 *m*/*s*^2^ is the gravitational acceleration and *l* is the radius of gyration which was 1.24 times the trochanteric height (Winter, 1979). The xCoM was regarded as an index of the CoM state that takes both the position and velocity into account.

### Statistics

The time to complete the task and variability in the foot placement were compared across conditions by using two-way repeated measures ANOVA (rmANOVA). The target size (small/large) and the target distance (short/long) were regarded as within-subject factors. Note that the side of the LED illumination (i.e., which leg was the lead/trail leg) was not regarded as a factor, and both of the left and right data were cumulated. For checking an assumption for rmANOVAs, we checked that the distributions of the data were approximately normal by using Q-Q plots. We did not explicitly perform normality tests because ANOVA is known to be robust to violation of normality, and multi-stage statistics would be a problem that is similar to multiple comparisons (Pituch and Stevens, 2016). The maximum CoP displacement and the CoM state (i.e., the position, velocity, and xCoM) were considered the control and the controlled parameters of the APA. In addition to the mean values for each condition, the within-condition within-participant standard deviations were also compared as indices of the variability in the APA parameters. Again, we used two-way rmANOVA to assess the APA parameters. The level of statistical significance for the rmANOVA was set to be α = 0.05. Eta squared was used as effect size of the rmANOVAs. Eta squared >0.01, 0.04, or 0.14 is interpreted as small, medium, and large effects, respectively. *Post-hoc* paired *t*-tests were carried out with the Bonfferroni-corrected significance level (*p* < 0.025). The effect size for the *post-hoc* tests was reported by using Cohen's *d*. Cohen's *d* > 0.2, 0.5, or 0.8 is interpreted as a small, medium, and large effects, respectively. All statistical analyses were performed using JASP ver. 0.14.1.0 (Eric-Jan Wagenmakers, Amsterdam, Netherlands).

## Results

**Figure 3:**
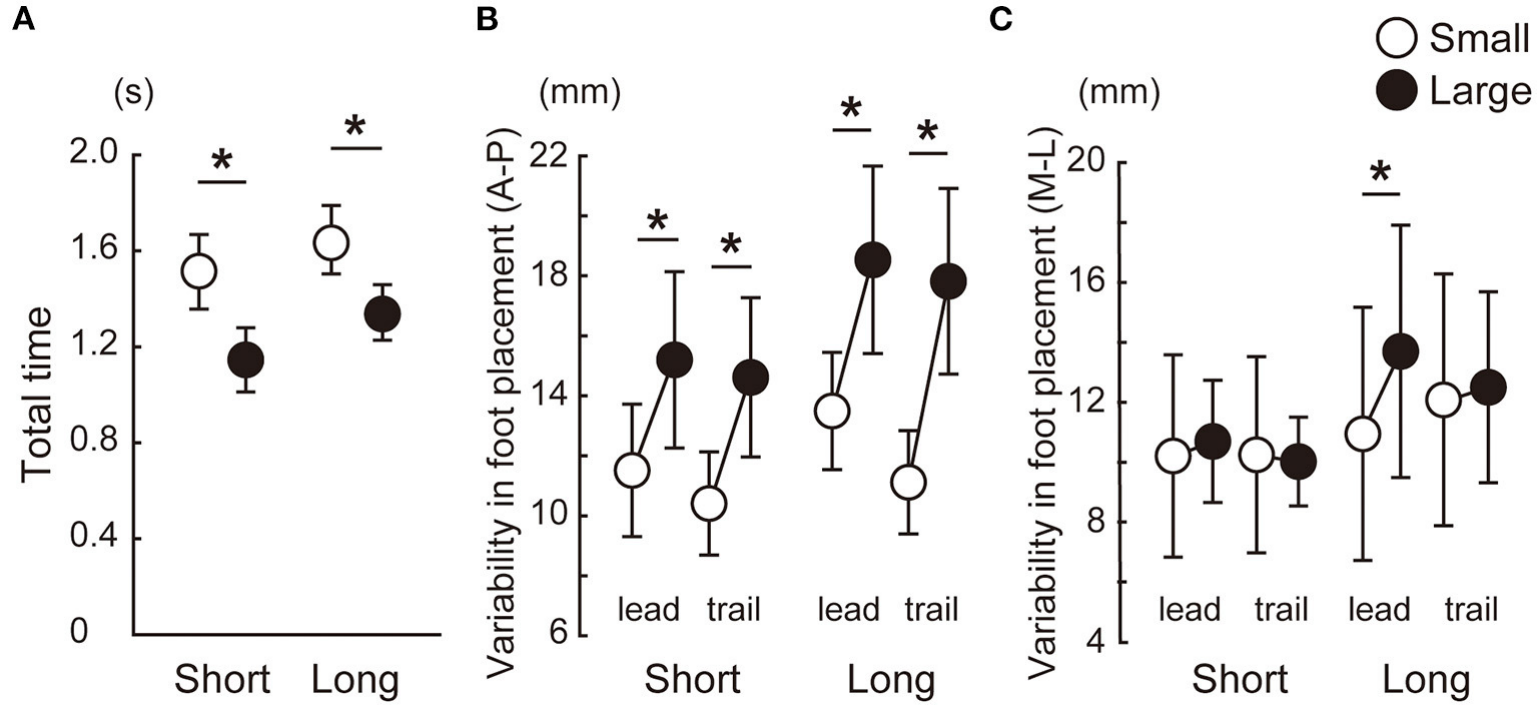
Speed-accuracy tradeoff in the forward stepping task in which participants pointed at targets with their great toes. Between-participant means and standard deviations are shown. The total step time was longer in the small target condition than in the large target condition **(A)**. The foot placement error was smaller in the small target conditions than in the large target conditions in both the anteroposterior axis **(B)** and mediolateral axis **(C)**. *Significant difference between the small and large target conditions (*p* < 0.025).

All the 16 participants completed the study. All the statistical results are shown as a table in a Supplementary Material. Figure 3 shows that the total step time was longer in the small target condition than in the large target condition. The variability in foot placement in the A-P direction was smaller in the small target condition than in the large target condition in both the leading and trailing legs and in both the short and long distance conditions. For the M-L axis, a significant difference was observed between the small and large conditions only for the leading leg in the long condition.

**Figure 4:**
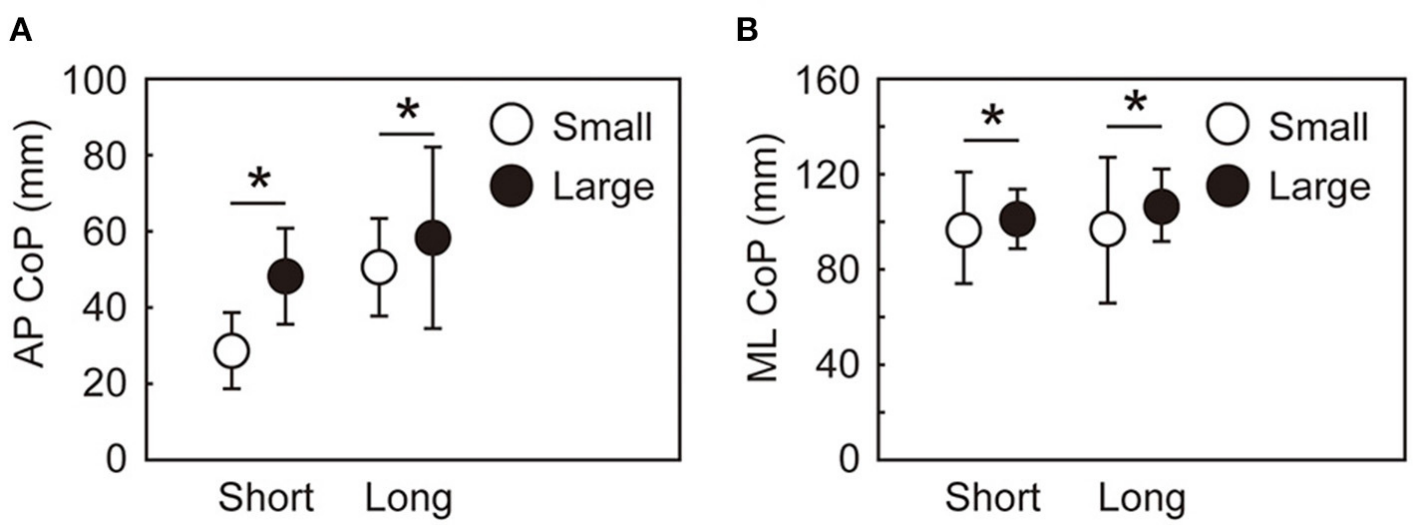
Maximum CoP displacement during the APA along with the AP **(A)** and ML **(B)** axes. Between-participant means and standard deviations are shown. *Indicates a significant difference between the small and large conditions (*p* < 0.025).

**Figure 5:**
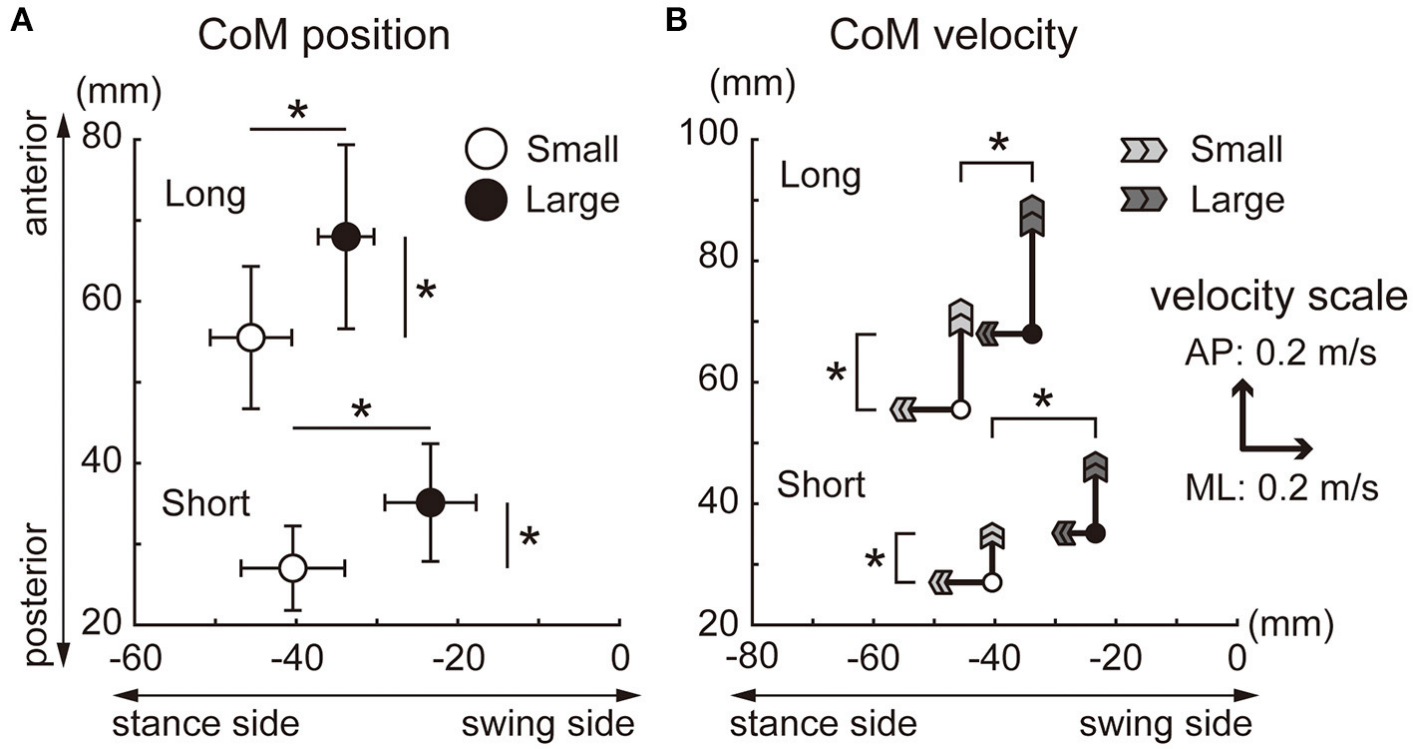
CoM position and velocity at takeoff of the leading leg. **(A)** CoM position. The locations of the circles indicate the average CoM position for each condition. The error bars indicate the between-participant standard deviations. **(B)** CoM velocity. The length of the arrows indicates the CoM velocity for each condition, and the size of the head of the arrows indicates the between-participant standard deviations. The starting point of the arrows indicates the CoM position (equivalent to **A**). *Indicates a significant difference in the CoM velocity (*p* < 0.025).

Significant main effects of target size and target distance were observed in the A-P and M-L CoP displacements, whereas the interaction between the target size and distance was not significant (Figure 4). The backward displacement of the CoP was smaller in the small condition than in the large condition [short: *t*(15) = 6.8, *p* < 0.001, *d* = 1.7; long: *t*(15) = 7.2, *p* < 0.001, *d* = 1.8]. The M-L CoP displacement toward the leading leg was larger in the small condition than in the large condition [short: *t*(15) = 4.9, *p* < 0.001, *d* = 1.2; long: *t*(15) = 2.9, *p* = 0.012, *d* = 0.7]. Figure 5 shows the mean CoM state at the time of takeoff of the leading leg. In the small target conditions, the CoM was positioned more posteriorly [short: *t*(15) = 6.3, *p* < 0.001, *d* = 1.6; long: *t*(15) = 7.0, *p* < 0.001, *d* = 1.8] and closer to the standing leg [short: *t*(15) = 10.1, *p* < 0.001, *d* = 2.5; long: *t*(15) = 9.7, *p* < 0.001, *d* = 2.4] than in the large conditions. The forward CoM velocity was smaller in the small condition than in the large condition [short: *t*(15) = 8.3, *p* < 0.001, *d* = 2.1; long: *t*(15) = 9.2, *p* < 0.001, *d* = 2.3]. In the M-L direction, the CoM moved faster toward the standing leg in the small target condition than in the large target condition [short: *t*(15) = 9.1, *p* < 0.001, *d* = 2.3; long: *t*(15) = 7.0, *p* < 0.001, *d* = 1.7].

**Figure 6:**
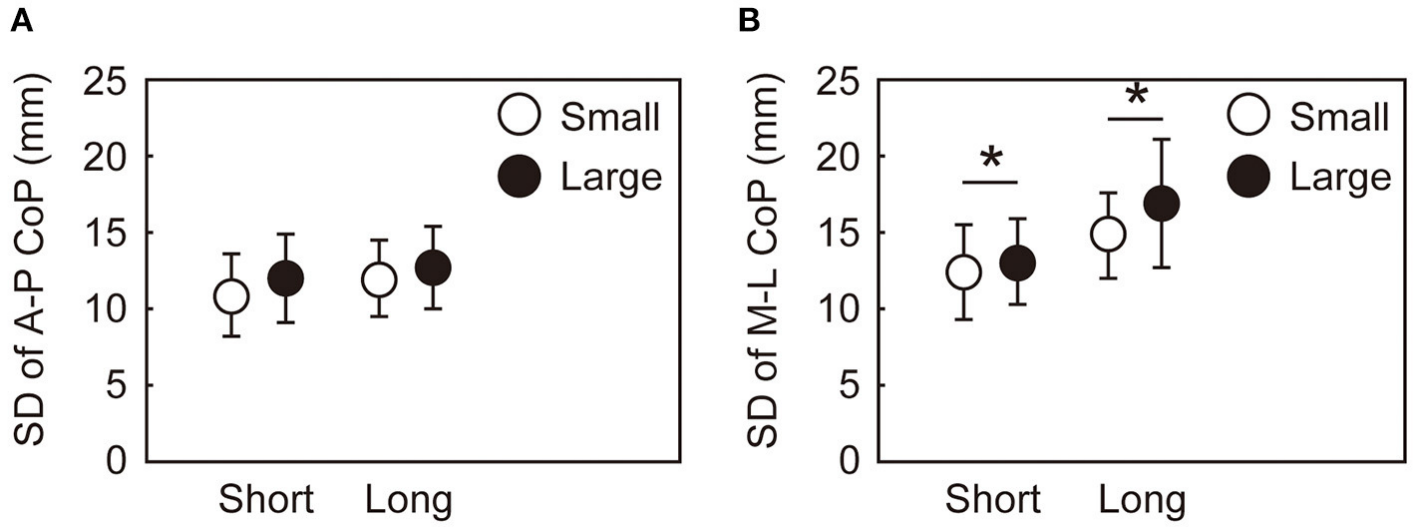
Within-participant standard deviations of the maximum CoP displacement during the APA along the AP **(A)** and ML **(B)** axes. The error bars show the between-participant standard deviations of the within-participant variability. *Indicates a significant difference between the small and large target conditions (*p* < 0.025).

**Figure 7:**
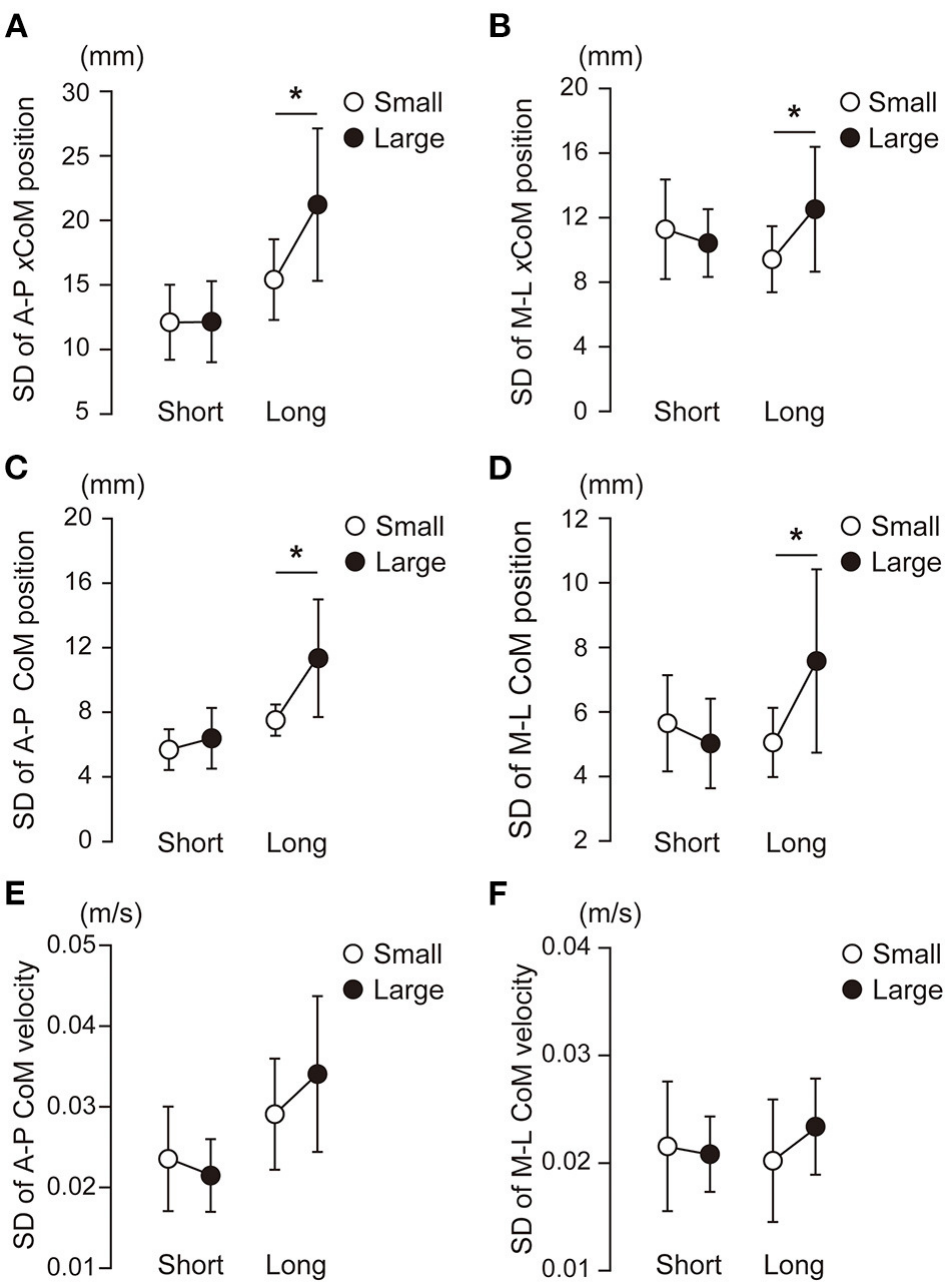
Within-participant variability in the center of mass (CoM) state at the instance of takeoff of the leading leg. The white and black circles show the within-participant standard deviations. The error bars show the between-participant standard deviations of the within-participant variability. The extrapolated center of mass (xCoM, **A,B**), CoM position **(C,D)** and CoM velocity **(E,F)** were analyzed. The left panels **(A,C,E)** show the CoM state in the anterior-posterior (A-P) axis, and the right panels **(B,D,F)** show the CoM state in the medial-lateral (M-L) axis. *Significant difference between the small and large target conditions (*p* < 0.025).

The variability in the CoP parameters during the APA and the CoM state at the time of takeoff of the leading leg also differed by the target size and target distance. Figure 6 shows the within-participant standard deviations of the CoP displacement during the APA. No significant interaction between target size and distance was observed in the variabilities in the A-P and M-L CoP displacement [A-P: *F*_(1, 15)_ = 0.175, *p* = 0.681, $\eta_{p}^{2}$ = 0.12; M-L: *F*_(1, 15)_ = 1.137, *p* = 0.303, $\eta_{p}^{2}$ = 0.07]. No main effect of target size nor target distance was observed for the variability in the A-P CoP displacement. The variability in the M-L CoP displacement was smaller in the small condition than in the large condition [*F*_(1, 15)_ = 21.25, *p* < 0.001, $\eta_{p}^{2}$ = 0.59]. The main effect of target distance was not significant on the variability in the M-L CoP displacement. The standard deviations of the CoM state at the time of takeoff of the leading leg are shown in Figure 7. The rmANOVA results revealed a significant interaction between the target size and target distance in A-P xCoM [*F*_(1, 15)_ = 12.9, *p* = 0.003, $\eta_{p}^{2}$ = 0.46]. The *post-hoc* analysis revealed that the standard deviation of the A-P xCoM was significantly smaller in the small target condition than in the large target condition in the long distance condition [*t*(15) = 3.8, *p* = 0.002, *d* = 0.96] but not in the short condition. A similar effect was observed in A-P CoM position variability, M-L xCoM variability and M-L CoM position variability (details are shown in a Supplementary Table 1).

## Discussion

The purpose of this study was to investigate the effect of required accuracy in a stepping initiation task on motor control during APAs. Our three hypotheses were supported by the results. First, our findings were in line with the speed-accuracy tradeoff principle applies to the step initiation task as well as the foot-reaching tasks performed in previous studies (Duarte and Latash, 2007; Bertucco and Cesari, 2010). When the target size was small, a longer time to complete the task and the smaller variability in the foot placement were observed. Second, in the small conditions, the APA was performed to prepare for a longer step time. The smaller backward CoP yielded a smaller forward displacement and velocity of the CoM than in the large conditions. For the M-L direction, the larger CoP displacement caused the CoM to be positioned more toward the standing limb. The third hypothesis was also supported: the variability in the CoP parameters during the APA and the CoM state at takeoff of the leading leg were lower in the small condition than in the large condition target condition when the target distance was long.

The total time was longer in the small target conditions than in the large target conditions due to the difference in the duration of the APA and swing phases, which is consistent with the speed-accuracy tradeoff principle. The reaction time was not affected by the target size. It is suggested that the time required for the sensorimotor processing before execution of the postural control and forward stepping was not influenced by the accuracy of the planned movement in our task. The speed-accuracy tradeoff is one of the most fundamental principles in motor control and was demonstrated by Fitts (1954). In the classic experiment, the movement time during a ballistic tapping task was found to be a function of the target size and distance. Because the principle applies to movements lasting for 200–800 ms, the principle is considered to describe the effectiveness of both feedback and feedforward control of human movement (Schmidt and Lee, 2011). Postural control is not an exception to the principle. Similar equations can be established for a foot-reaching task or standing postural control during an arm reaching task (Duarte and Latash, 2007; Bertucco et al., 2013). Our study adds another empirical finding that the movement time might be planned and executed as a function of the target size and distance in a step initiation task.

In stepping tasks such as the foot-pointing task or step initiation task, the CoM state must be appropriately set before the takeoff of the leading leg by controlling the CoP displacement during an APA. Human stepping is modeled as an inverted pendulum, where the whole-body CoM moves around the CoP under the influence of gravity (Winter, 1995; Lyon and Day, 1997; Gage et al., 2004; Manoj and Andy, 2006; Yiou et al., 2016b). In this model, the CoM state during the stepping phase is determined as the time evolution of the initial CoM state (Lyon and Day, 1997; Pai and Patton, 1997). Because of the related dynamics, if an individual wants to have a long swing phase, the CoM must move close to the standing leg to stabilize the CoM (Caderby et al., 2014; Yiou et al., 2016a). The small conditions in the present study, compared with the large conditions, yielded a smaller backward CoP displacement and a larger M-L CoP displacement toward the side of the standing limb, which supports our second hypothesis. These CoP displacements caused the CoM to be positioned more on the side of the standing leg, with a smaller forward velocity, which was also confirmed by our results. These observations support the idea that the motor control for slow stepping that was planned to achieve accurate stepping was started with CoM control during the APA.

The inter-trial variability in the CoM state also suggests task-relevant postural control for accurate stepping, supporting our third hypothesis. Given that the sensorimotor signals are corrupted by noise whose variance increases with the size of the signal (Harris and Wolpert, 1998), reducing the amplitude of APA might lead to the observed smaller variabilities in CoP and CoM parameters during APA. However, the results of the amplitude of the CoP and CoM parameters could not explain the results of the variability of those parameters. We observed smaller variability in the M-L CoP displacement during the APA and in the A-P and M-L CoM and xCoM positions in the long distance condition, but not in the short distance condition. Because passive dynamics dominate the CoM movements during the stepping phase (Bottaro et al., 2005), large variability at the time of takeoff will lead to larger variability at the end of the step, which leads to inaccurate step placement. This effect is larger for steps with longer step lengths. The participants might take these factors into account during the swing phase, leading to accurate control of the CoM state during the APA only in the long conditions. The results of the present study indicate task-relevant control of the CoM during APAs to achieve the required accuracy in stepping.

APAs are a type of predictive control during step initiation and involve both propulsion (Brenière et al., 1987; MacKinnon et al., 2007) and postural stability (Jian et al., 1993; McIlroy and Maki, 1999; Singer et al., 2013; Yiou et al., 2016a). Many studies have indicated that various task requirements are related to APAs. APAs change in response to task requirements such as step length (Brenière et al., 1987; Zettel et al., 2002), gait speed (Caderby et al., 2014), the existence of perturbations (Schlenstedt et al., 2017), step direction (Tateuchi et al., 2011) or obstacles (Yiou et al., 2016a). Duarte and Latash (Duarte and Latash, 2007) reported that the CoP amplitude and its variability are affected by the target size in a forward foot-reaching task. The present study has provided new insight regarding variability in CoP displacement and the CoM state, as well as their mean values, showing that they are task-relevantly regulated during APAs according to the required stepping accuracy.

The results of this study suggest that the motor control for accurate stepping begins before the actual leg movements start. In the fields of sports training, players, coaches, and sports scientists should focus not only on noticeable leg movements but also on the invisible APA. A previous study reported that inconsistent APA was potentially related to the risk of injury and suggested an importance of the systematic tests of APA on high-level athletes such as rugby players (Wang et al., 2018). Another study demonstrated that the CoP displacement and ground reaction forces during APA before forward stepping were affected by psychological pressure (Sasaki and Sekiya, 2018). These studies suggest possibility that APA may be a sensitive indicator that reflects injury risk and sports performance. Importantly, the variability in the APA cannot be assessed by looking at a single trial. Technical assistance for assessing and visualizing the APA parameters and their variability would be useful.

Several limitations should be noted. First, we only recruited healthy male participants that could not reflect a whole of young population. Some studies reported sex differences in the prevalence of posture-related deficit such as back pain (Schneider et al., 2006) and APA parameters tested in laboratory experiments (Bussey et al., 2018). If we want to extend our knowledge to a clinically relevant way, future studies would be needed based on a wider range of subjects. Second, since the task constraints in this study were the target size, the CoM state was not explicitly restricted in the task. For aiming at the target on the floor, the participants might change the CoM position, and/or they might control the position of the foot relative to the CoM. This is not the same situation as stepping onto a small stepping stone on a pond, where one has to accurately control the CoM state to prevent oneself from falling into the water. Third, because this study tested healthy young male participants only, clinical relevance would subject to future studies. Previous studies reported that aging and neuropathological diseases change the characteristics of APAs (Lin et al., 2016; Cohen et al., 2017). Although the variability in the CoM state during step initiation could potentially be related to fall risks or other age-related symptoms, there is no knowledge about this issue. Additional research investigating the variability in CoM control during APAs in elderly people and people with various diseases would be relevant for a better assessment of fall risks.

To summarize, we had three findings. First, stepping onto the small targets took a longer time compared to the large target conditions, which follows the speed-accuracy tradeoff principle. Second, in the small condition, the smaller backward CoP displacement and larger M-L CoP displacement toward the standing limb can be regarded as an appropriate preparatory action for a slower step. Last, not only the mean values but also the variability in the CoP displacement and the variability the CoM state were smaller in the small condition than in the large condition when the target distance was long.

## Data Availability Statement

The raw data supporting the conclusions of this article will be made available by the authors, without undue reservation.

## Ethics Statement

The studies involving human participants were reviewed and approved by Graduate School of Integrated Arts and Sciences, Hiroshima University. The patients/participants provided their written informed consent to participate in this study.

## Author Contributions

HY and MS contributed to the design and implementation of the research, the analysis of the results, and the manuscript's writing. All authors contributed to the article and approved the submitted version.

## Conflict of Interest

The authors declare that the research was conducted in the absence of any commercial or financial relationships that could be construed as a potential conflict of interest.

## Publisher's Note

All claims expressed in this article are solely those of the authors and do not necessarily represent those of their affiliated organizations, or those of the publisher, the editors and the reviewers. Any product that may be evaluated in this article, or claim that may be made by its manufacturer, is not guaranteed or endorsed by the publisher.
